# Agreement Analysis between Sonographic Estimates and Birth Weight, by the WHO and Intergrowth-21st Tables, in Newborns of Diabetic Mothers

**DOI:** 10.1055/s-0040-1719146

**Published:** 2021-01-29

**Authors:** Marcus Vinícius Rodrigues de Souza, Lívia Pinto e Fróes, Pedro Afonso Cortez, Márcio Weissheimer Lauria, Regina Amélia Lopes de Aguiar, Kamilla Maria Araújo Brandão Rajão

**Affiliations:** 1 Universidade Federal de Minas Gerais, Belo Horizonte, MG, Brazil; 2 Universidade Metodista de São Paulo, São Bernardo do Campo, São Paulo, SP, Brazil

**Keywords:** abdominal circumference, estimated fetal weight, birth weight, gestational diabetes, growth charts, circunferência abdominal, peso fetal estimado, peso ao nascer, diabetes gestacional, gráficos de crescimento

## Abstract

**Objective** To analyze the agreement, in relation to the 90th percentile, of ultrasound measurements of abdominal circumference (AC) and estimated fetal weight (EFW), between the World Health Organization (WHO) and the International Fetal and Newborn Growth Consortium for the 21 ^st^ Century (intergrowth-21 ^st^ ) tables, as well as regarding birth weight in fetuses/newborns of diabetic mothers.

**Methods** Retrospective study with data from medical records of 171 diabetic pregnant women, single pregnancies, followed between January 2017 and June 2018. Abdominal circumference and EFW data at admission (from 22 weeks) and predelivery (up to 3 weeks) were analyzed. These measures were classified in relation to the 90th percentile. The Kappa coefficient was used to analyze the agreement of these ultrasound variables between the WHO and intergrowth-21 ^st^ tables, as well as, by reference table, these measurements and birth weight.

**Results** The WHO study reported 21.6% large-for-gestational-age (LGA) newborns while the intergrowth-21 ^st^ reported 32.2%. Both tables had strong concordances in the assessment of initial AC, final AC, and initial EFW (Kappa = 0.66, 0.72 and 0.63, respectively) and almost perfect concordance in relation to final EFW (Kappa = 0.91). Regarding birth weight, the best concordances were found for initial AC (WHO: Kappa = 0.35; intergrowth-21 ^st^ : Kappa = 0.42) and with the final EFW (WHO: Kappa = 0.33; intergrowth- 21 ^st^ : Kappa = 0.35).

**Conclusion** The initial AC and final EFW were the parameters of best agreement regarding birth weight classification. The WHO and intergrowth-21 ^st^ tables showed high agreement in the classification of ultrasound measurements in relation to the 90th percentile. Studies are needed to confirm whether any of these tables are superior in predicting short- and long-term negative outcomes in the LGA group.

## Resumo

**Objetivo** Analisar a concordância, em relação ao percentil 90, das medidas ultrassonográficas da circunferência abdominal (CA) e peso fetal estimado (PFE), entre as tabelas da Organização Mundial de Saúde (OMS) e do *International Fetal and Newborn Growth Consortium for the 21 ^st^ Century* integrowth-21 ^st^ , bem como em relação ao peso ao nascer em fetos/recém-nascidos de mães diabéticas.

**Métodos** Estudo retrospectivo com dados de prontuários de 171 gestantes diabéticas, com gestações únicas, seguidas entre Janeiro de 2017 e Junho de 2018. Foram analisados dados da CA e do PFE na admissão (a partir de 22 semanas) e no pré-parto (até 3 semanas). Essas medidas foram classificadas em relação ao percentil 90. O coeficiente Kappa foi utilizado para analisar a concordância entre as tabelas da OMS e Intergrowth-21 ^st^ , assim como, por tabela de referência, entre as medidas e o peso ao nascer.

**Resultados** O estudo da OMS relatou 21,6% dos recém nascidos grandes para a idade gestacional (GIG) enquanto que o estudo do intergrowth-21 ^st^ relatou 32,2%. Ambas as tabelas tiveram fortes concordâncias na avaliação da CA inicial e final e PFE inicial (Kappa = 0,66, 0,72 e 0,63, respectivamente) e concordância quase perfeita em relação ao PFE final (Kappa = 0,91). Em relação ao peso ao nascer, as melhores concordâncias foram encontradas para a CA inicial (OMS: Kappa = 0,35; intergrowth-21 ^st^ : Kappa = 0,42) e com o PFE final (OMS: Kappa = 0,33; intergrowth-21 ^st^ : Kappa = 0,35).

**Conclusão** A CA inicial e o PFE final foram os parâmetros de melhor concordância em relação à classificação do peso ao nascer. As tabelas da OMS e intergrowth-21 ^st^ mostraram alta concordância na classificação das medidas ultrassonográficas em relação ao percentil 90. Estudos são necessários para confirmar se alguma dessas tabelas é superior na previsão de resultados negativos a curto e longo prazo no grupo GIG.

## Introduction

Diabetes mellitus (DM) is a major public health problem, and its prevalence has increased over the years, including in women of childbearing age, because of the epidemic of overweight and obesity in the world. 1 In Brazil, this prevalence varies from 1.3%, in the age group between 18 and 24 years, to 4.6%, in the 35 to 44 years age group; thus, Brazil is ranked 5 ^th^ in the DM prevalence among adults in the world. 2 3 4

Regarding diabetes in pregnancy, one in six women giving birth in the world has hyperglycemia, of which 84% are due to gestational diabetes mellitus (GDM). 5 The Brazilian Gestational Diabetes Study has documented a prevalence of 7.6% of GDM using the 1999 World Health Organization (WHO) criteria. 6 However, according to the criteria of the International Association of Diabetes in Pregnancy Study Group (IADPSG), the GDM prevalence in the Brazilian public health care system has reached 18%. 7 8

The use of ultrasound scanning is fundamental to monitor fetal growth, especially when there is an increased risk of large for gestational age (LGA), as it occurs with diabetic mothers' fetuses. 9 Some of the parameters used for this estimate are the abdominal circumference (AC) and the estimated fetal weight (EFW). 9 Abdominal circumference is considered the earliest and, therefore, the most sensitive parameter in the evaluation of fetal macrosomia, reflecting liver growth abnormalities. 10 Estimated fetal weight is an indirect measurement calculated with formulas that use multiple biometric parameters, and it is subject to a higher percentage of errors. 11 Detecting fetal growth abnormalities is very important for therapeutic decisions, both to correct predisposing factors and to predict the type and moment of delivery. 12

Nevertheless, the occurrence of LGA is also associated with obstetric and neonatal adverse outcomes, such as increased cesarean delivery rates, neonatal hypoglycemia, jaundice, 5-minute Apgar score < 7, higher stillbirth rate, and respiratory distress. 13 14 Some studies have also pointed out that the consequences of being born LGA may go beyond the neonatal period, increasing the risk of precocious puberty, childhood obesity, and metabolic syndrome in childhood and adulthood. 15 16

Until recently, references of ultrasound assessment of fetal growth have been based on single-center studies, with a few measurements from North American populations with low ethnic variability. 17 To create more representative references of the world population, two studies were conducted with representativeness of the Brazilian population: one by WHO and the other by the International Fetal and Newborn Growth Consortium for the 21 ^st^ Century (intergrowth-21 ^st^ ). 18 19

Given the divergences observed in the clinical practice between the LGA classifications by the WHO's and intergrowth-21 ^st^ 's studies—which resulted in an increase in LGA cases after the adoption of these reference tables—and the apparent divergence between the EFG and the real birth weight, we proposed the development of the present study. Our aim was (i) to analyze the level of agreement of these two LGA classifications in a population of diabetic pregnant women and (ii) to compare the birth weight results in relation to the ultrasound variables during the third trimester of pregnancy, to determine whether there was any disagreement between these results.

## Methods

### Participants

This is a retrospective cohort study based on a survey of medical records of 171 diabetic pregnant women followed up by the Obstetrics and Endocrinology Services of one tertiary Hospital from Belo Horizonte, in the state of MG, Brazil. The participants received care between January 2017 and June 2018. Data were collected between August and November 2018. This study was approved by the Research Ethics Committee (CAAE: 50724015.3.0000.5149), and all participants signed the informed consent form.

**Fig. 1:**
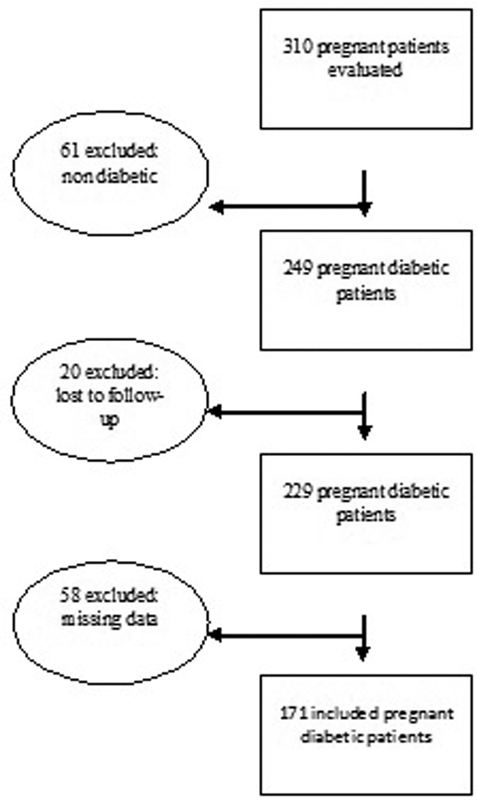
Participants exclusion and inclusion criteria.

First, we obtained the record from all the patients representing 310 diabetic and non diabetic pregnant patients. Then, we applied the exclusion criteria: a) non diabetics; b) patients lost to follow-up; c) missing data on patient medical records to include in the study. We excluded 139 patients. All the other 171 participants were included in the present study and attended the following inclusion criteria: pregnant women diagnosed with pregestational or gestational diabetes, with single pregnancies, aged ≥18 years, regularly monitored during the previously mentioned period, with available ultrasound measurements (the availability of EFW at the first ultrasound scan and the data on birth weight and gestational age at birth were mandatory), and those women who consented to participate in the research. Twin pregnancies were excluded because they were not represented in the WHO's and intergrowth-21 ^st^ 's studies. The inclusion and exclusion criteria are shown in Fig. 1 .

The clinical variables reported were maternal age (years), type of diabetes (gestational or pregestational), classification of pregestational diabetes cases (type 1 DM [T1D], type 2 DM [T2D], overt diabetes, or diabetes due to other causes), gestational age at birth (weeks), and birth weight (g). Gestational age was calculated by the Obstetrics Service based on the date of women's last period or the first ultrasound scan available, as recommended by the American College of Obstetricians and Gynecologists. 20

All pregnant women who had no previous diagnosis of diabetes were screened with a fasting glucose test up to 20 weeks of gestation. When the initial fasting blood glucose was < 92 mg per deciliter [5.11 nmol per liter], the diagnosis of GDM was based on the IADPSG recommendations, endorsed by the American Diabetes Association (ADA), using the 75G-OGTT (oral glucose tolerance test) at 24 to 28 weeks of gestational age (fasting ≥ 92 mg per deciliter [5.1 nmol per liter] or 1 hour post dextrosol ≥180 mg per deciliter [10 nmol per liter] or 2 hours post dextrosol ≥ 153 mg per deciliter [8.5 nmol per liter]). 7 21 When the fasting glucose test result before 20 weeks was between 92 to 125 mg per deciliter [5.1 to 6.9 nmol per liter], with a second confirmatory sample, early GDM was diagnosed, following the protocol recommended by the Pan American Health Organization (PAHO). 2 The diagnosis of pre-gestational DM was defined according to the patient's report of previous diagnosis and treatment, or defined according to ADA recommendations, based on the following criteria: presence of classic symptoms of hyperglycemia and random glucose ≥ 200 mg per deciliter [11.1 nmol per liter] or asymptomatic patient with two altered tests: fasting glucose (8 hours) ≥ 126 mg per deciliter [7 nmol per liter] and/or glycohemoglobin (HbA1c) ≥ 6.5% (using a method approved by the National Glycohemoglobin Standardization Program) and/or 75 g-OGTT ≥ 200 mg per deciliter [11.1 nmol per liter]. 21

During prenatal care, EFW ≥ 90 ^th^ percentile (p90) was considered LGA. During the newborn assessment, LGA was applied to birth weight, according to gestational age, as ≥ p90. 22 This classification was performed according to the WHO's (for gender and gestational age) and intergrowth-21 ^st^ 's (for gestational age) studies. 18 19 The sonographic measurements used in the present study were obtained by trained professionals from the obstetrics service. The AC and EFW values obtained in the first ultrasound performed after 22 weeks of gestation were reported as “initial” values, and the same variables measured in the last ultrasound performed up to three weeks before delivery were reported as “final” values and considered for analysis, according to data availability in the medical records. The EFW was calculated in the obstetrics service according to the recommendations previously published by Hadlock et al. (1984). 23 These measurements were categorized as < p90 or ≥ p90 for comparison with the same birth weight percentiles.

## Statistical Analysis

A statistical analysis was performed using MedCalc version 19.1 (MedCalc Software Ltd, Ostend, Belgium). The Shapiro-Wilk normality test was applied to quantitative variables to verify normality distribution (for maternal age: W = 0.98; *p* = 0.0099; for gestational age at birth: W = 0.79, *p* < 0.0001; and for birth weight: W = 0.96; *p* = 0.0001). Since none of them had a normal distribution, these variables were reported as median and interquartile range (p25–75).

Estimated fetal weight, AC, and birth weight were categorized as < p90 or ≥ p90 and reported as proportions. The Kappa coefficient was used to analyze clinimetric evidence considering the agreement of results in relation to ultrasound measurements between the WHO's and intergrowth-21 ^st^ 's classifications. Then, the Kappa coefficient was also used to analyze the agreement, within the same classification table, between the sonographic variables (AC and EFW, at initial and final ultrasonography) in relation to birth weight. The Kappa coefficient between 0 and 0.2 is considered weak; between 0.21 and 0.4, reasonable; between 0.41 and 0.6, moderate; between 0.61 and 0.8, strong; and between 0.81 and 1, almost perfect. 24

## Results

We analyzed data from the medical records of 171 pregnant women who met the inclusion criteria. Of this total, 65 (38.0%) participants had pregestational DM and 106 (62.0%) GDM, with a median age of 33 years (29–37 years). Regarding the pregestational DM group, 21.5% ( *n* = 14) were T1D, 46.2% ( *n* = 30) T2D, 30.8% ( *n* = 20) overt diabetes, and 1.5% ( *n* = 1) had other cause of DM (pancreatic). The median gestational age at birth was 38 weeks (37–38 weeks). The median birth weight was 3,185 g (2,757–3,519 g). Considering the LGA outcome according to the WHO classification, 37 cases (21.6%) were detected; according to the intergrowth-21 ^st^ classification, 55 cases were detected (32.2%).

The first ultrasound scans were performed at a median of 30 weeks (21–36 weeks), and the final ultrasound scans at a median of 35 weeks (26–39 weeks). Descriptive analysis comparing the two reference tables used in this study regarding AC, EFW and birth weight is shown in Table 1 . The agreement of classifications was considered strong for initial AC, final AC, initial EFW and birth weight, and almost perfect for the final EFW. Table 2 shows the results.

**Table 1:** Classification of sonographic variables and birth weight, based on the WHO's and intergrowth-21 ^st^ 's studies

	WHO		Intergrowth-21 ^st^		Total
	≥ p90	< p90	≥ p90	< p90	
	N (%)	N (%)	N (%)	N (%)	
Initial AC	40 (27.4)	106 (72.6)	61 (41.8)	85 (58.2)	146 (100.0)
Initial EFW	66 (38.6)	105 (61.4)	79 (46.2)	92 (53.8)	171 (100.0)
Final AC	30 (25.6)	87 (74.4)	42 (35.9)	75 (64.1)	117 (100.0)
Final EFW	37 (28.2)	94 (71.8)	40 (30.5)	91 (69.5)	131 (100.0)
Birth weight	37 (21.6)	134 (78.4)	55 (32.2)	116 (67.8)	171 (100.0)

Abbreviations: AC, abdominal circumference; EFW, estimated fetal weight; WHO, World Health Organization.

**Table 2:** Agreement analysis between sonographic variables based on the WHO's and intergrowth-21 ^st^ 's studies

	Agreement with ≥ p90 N (%)	Agreement with < p90 N (%)	Disagreement N (%)	Kappa	CI95%
Initial AC	39 (26.7)	84 (57.5)	23 (15.8)	0.66	[0.54;0.78]
Initial EFW	57 (33.3)	83 (48.6)	31 (18.1)	0.63	[0.52;0.75]
Final AC	29 (24.8)	74 (63.2)	14 (12.0)	0.72	[0.59;0.85]
Final EFW	36 (27.5)	90 (68.7)	5 (3.8)	0.91	[0.83;0.99]
Birth weight	37 (21.6)	116 (67.8)	18 (10.5)	0.74	[0.62;0.85]

Abbreviations: AC, abdominal circumference; CI, confidence interval; EFW, estimated fetal weight; WHO, World Health Organization.

Considering birth weight as a reference measurement, we analyzed the agreement of this measure with the ultrasound parameters, AC and EFW. Table 3 presents the analysis made according to the WHO classification, and Table 4 displays the results based on the intergrowth-21 ^st^ classification. The best agreement results were obtained for the initial AC and final EFW parameters, both in the WHO and intergrowth-21 ^st^ classifications, and they are considered reasonable. However, the Kappa value was higher in relation to the reference measurements obtained by the intergrowth-21 ^st^ classification.

**Table 3:** Agreement analysis between sonographic variables and birth weight based on the WHO classification

	Agreement with ≥ p90 N (%)	Agreement with < p90 N (%)	Disagreement N (%)	Kappa	CI95%
Initial AC	19 (13.0)	91 (62.3)	36 (24.7)	0.35	[0.18;0.52]
Initial EFW	21 (12.3)	89 (52.0)	61 (35.7)	0.18	[0.04;0.32]
Final AC	11 (9.4)	71 (60.7)	35 (29.9)	0.19	[0.00;0.38]
Final EFW	17 (13.0)	80 (61.1)	34 (25.9)	0.33	[0.15;0.51]

Abbreviations: AC, abdominal circumference; CI, confidence interval; EFW, estimated fetal weight; WHO, World Health Organization.

**Table 4:** Agreement analysis between sonographic variables and birth weight based on the intergrowth-21 ^st^ classification

	Agreement with ≥ p90 N (%)	Agreement with < p90 N (%)	Disagreement N (%)	Kappa	CI95%
Initial AC	35 (24.0)	71 (48.6)	40 (27.4)	0.42	[0.27;0.57]
Initial EFW	36 (21.1)	73 (42.7)	62 (36.2)	0.18	[0.04;0.32]
Final AC	22 (18.8)	58 (49.6)	37 (31.6)	0.30	[0.12;0.48]
Final EFW	23 (17.6)	71 (54.2)	37 (28.2)	0.35	[0.18;0.52]

Abbreviations: AC, abdominal circumference; CI, confidence interval; EFW, estimated fetal weight.

## Discussion

This unprecedented study analyzed—in a sample of 171 diabetic pregnant women treated in a Brazilian public tertiary care service—the agreement between ultrasound parameters (AC and EFW) and birth weight, comparing of WHO's and intergrowth-21 ^st^ 's LGA classifications, which have representativeness of Brazilian pregnant women. We found a high agreement between the two studies regarding the measurements of initial AC, final AC, and initial EFW, and an almost perfect agreement with the final EFW. This study was justified because it approaches a population at risk of excessive fetal growth, and it is based on the observation of the alarming number of referrals of LGA cases based on the first fetal ultrasound scan, even though glycemic control and other lifestyle changes can positively affect the reduction of negative gestational and neonatal clinical outcomes.

The EFW measurement is extremely important in clinical practice, especially for the management of high-risk pregnancies, such as diabetic pregnant women. 18 The final EFW was one of the measurements with the highest agreement in relation to birth weight (Kappa = 0.33; CI95% [0.18;0.52] for WHO; Kappa = 0.35; CI95% [0.15;0.51] for intergrowth-21 ^st^ ). Barel et al. 25 found that the accuracy of these measurements has decreased in weight extremes (< 2,000 g or > 4,000 g)—which may have resulted in such poor agreement with the initial EFW—, and more LGA cases were detected in the first ultrasound scan. In a retrospective cohort study with macrosomic fetuses (from 4,000 g to ≥ 4,750 g), Zafman et al. 26 found overestimated weight values by ultrasound scans in more than 50% of cases, especially in the groups with higher fetal weight, as high as reported in this paper. In another cohort study, in which 32-week-old ultrasound scans of 521 diabetic pregnant women were analyzed, the authors found that EFW had a reasonable sensibility (80.3%) and a high negative predictive value (96%), but a low positive predictive value (38%) in LGA detection. 27

In addition to weight extremes, late gestational ages and obesity itself can reduce the accuracy of ultrasound results in diabetic pregnant women compared with low-risk pregnant women. 27 However, as it is a calculated indirect measurement, the EFW has an estimated margin of error of 10 to 15%. 28 Thus, all these factors point us to look differently to the measurements of this population.

Another measurement that showed one of the best agreement regarding birth weight percentiles was the initial AC (Kappa = 0.35; CI95% [0.18;0.52] for WHO; Kappa = 0.42; CI95% [0.15;0.51] for intergrowth-21 ^st^ ). In a Chinese prospective multicenter study of 8,272 DM cases and 729 pre-gestational DM cases, Yan et al. showed that the growth rates assessed by means of AC were higher in the GDM and pre-gestational DM groups with macrosomic babies than in non-macrosomic groups, with statistically significant differences between groups older than 22 weeks of gestation ( *p* = 0.001). 9 In another cohort in South Africa, Macauley et al. 29 followed up 741 women with serial ultrasound measurements and showed that AC among pregnant women with GDM (24–28 weeks) was significantly higher in all measurements, from 14 weeks to 38 weeks of gestational age, especially between 27 and 32 weeks ( *p* < 0.001), regardless of BMI (body mass index). 29 In addition, Brand et al. 30 showed that, in a population of South Asian and English descendants, fetal growth accelerates after 24 weeks of age until birth in pregnant women with GDM, even before they are diagnosed with GDM. 30 These findings reinforce the importance of this measurement as an early warning of excessive fetal growth.

Differences between the WHO's and intergrowth-21 ^st^ 's studies are evident. For example, in the LGA classification at birth, 21.6% of cases ( *n* = 37/170) were detected by the WHO and 32.2% ( *n* = 55/170) by intergrowth-21 ^st^ . With a population similar to the one investigated in the present study, the WHO used Hadlock's formula to calculate EFW, while intergrowth-21 ^st^ created its own formula to calculate the EFW using only AC and head circumference. Further, starting at 25 weeks, EFW's p90 cutoff in intergrowth-21 ^st^ 's is lower than in WHO's (for example: for 38 weeks, cutoffs would be 3,540 g in intergrowth-21 ^st^ 's and 3,616 g in WHO's). Concerning AC, this difference can be observed as early as 14 weeks (in the same example, at 38 weeks, AC's p90 cutoff is 356.4 mm in the intergrowth-21 ^st^ 's study and 364 mm in the WHO's study). 31

In addition, the populations included in the studies were different. The WHO's study included 1,387 healthy pregnant women with good socioeconomic, environmental and nutritional status, aged 18 to 40 years, in single pregnancies, and from 10 countries (Brazil, Germany, Argentina, Congo, Norway, Thailand, India, France, and Egypt). In Brazil, the University of Campinas (Campinas, SP) was the center that participated in the study, with a total of 148 women. The pregnancies had an average duration of 39 weeks. Significant differences were observed in EFW among countries, both in the lowest (3.5%) and the highest percentiles (4.5%). Maternal age, weight and parity contributed to these differences. These variations between countries and the number of countries represented in it were limiting conditions for the widespread use of this scale, according to the authors. 18

The intergrowth-21 ^st^ 's study included, for the purpose of generating growth tables, 1,556 healthy, well-nourished pregnant women with low risk of maternal and perinatal adverse events, between 14 weeks of gestation and with babies up to 2 years of age, from 8 countries (Brazil, China, United Kingdom, Oman, Italy, Kenya, India, and United States of America). Brazil was represented by the Federal University of Pelotas (Pelotas, RS). This study, like WHO's, worked as the prescriptive concept of growth, in which healthy populations have similar growth patterns, but did not perform statistical analysis between the populations of the different countries included. 19

Some studies have compared LGA and AC detection rates above p90 between the intergrowth-21 ^st^ and reference tables generated by population studies in different countries. In an Australian retrospective hospital-based cohort study with 2,966 unselected pregnant women, 16.5% of newborns were classified as LGA after 33 weeks of gestation using intergrowth-21 ^st^ 's p90. 32 This represented 66% more cases detected when compared with the reference growth tables for the country's population. Multivariate analysis identified two independent predictors: the presence of pregestational diabetes and high pregestational BMI. 32 A French cohort study conducted by Heude et al. 33 analyzed 14,607 single pregnancies, of which 34% were at low risk according to the inclusion criteria of intergrowth-21 ^st^ , with 5 to 10% of gestational diabetes cases. The authors reported 16.7% of AC cases ≥ p90 according to intergrowth-21 ^st^ , compared with 7.1% of cases according to French population's specific growth curves. These findings, however, were similar between unselected population and low-risk cases, both in the second and third trimester. 33 Brazil has not had any comprehensive study to develop a representative table of its population. A recent meta-analysis with studies of the Brazilian population has found between 4.1 and 30.1% cases of excessive fetal growth. The criteria adopted varied among the different studies, which accepted as excessive fetal growth birth weight ≥ 90 and macrosomia while birth weight ≥ 4,000 g. 12 Thus, it is necessary, in our country, with its wide territorial extension and ethnic variety, to carry out a study to establish national clinimetric evidence for Brazilian reference curves. 34

There are several limitations to our work. This is a retrospective study based on medical record data, with a limited number of participants, some data loss, and a poor description of the procedures and results that could contribute to a more complete analysis. In addition, patient admission was not homogeneous and, sometimes, even late, limiting the early appropriate execution of interventions and even ultrasound measurements, especially in pregnant women with pregestational DM. As a research agenda, future studies may investigate, prospectively and in a multicenter way, the Brazilian population to determine whether the differences between these two studies, regarding LGA detection, affects neonatal outcomes, such as complications and mortality, so they can be safely inserted into clinical practice.

## Conclusion

In a Brazilian population of diabetic pregnant women, we found strong agreement for ultrasound measurements of AC and EFW between the WHO's and intergrowth-21 ^st^ 's studies, with a tendency to overestimate weight based on the first ultrasound scans. The initial AC and EFW at the last ultrasound scan were the best agreement parameters for birth weight, as corroborated by other studies in the international literature, with a better agreement obtained by the intergrowth-21 ^st^ classification. Higher LGA detection rates were observed in intergrowth-21 ^st^ 's tables. Further studies are needed to define the study that better applies to our population, objectively analyzing outcomes such as short and long-term neonatal and postnatal complications in relation to LGA fetuses.
